# Gender differences in BaYaka forager sleep-wake patterns in forest and village contexts

**DOI:** 10.1038/s41598-021-92816-6

**Published:** 2021-07-01

**Authors:** Erica Kilius, David R. Samson, Sheina Lew-Levy, Mallika S. Sarma, Ujas A. Patel, Yann R. Ouamba, Valchy Miegakanda, Lee T. Gettler, Adam H. Boyette

**Affiliations:** 1 Department of Anthropology, University of Toronto Mississauga, Mississauga, L5L1C6 Canada; 2 Department of Psychology, Simon Fraser University, Burnaby, V5A1S6 Canada; 3 Department of Archaeology and Heritage Studies, Aarhus University, 8270 Højbjerg, Denmark; 4 School of Medicine, Johns Hopkins University, Baltimore, 21287 USA; 5 Ecole Nationale Supérieure d’Agronomie et de Foresterie, Université Marien N’Gouabi, Brazzaville, Republic of the Congo; 6 Laboratoire National de Santé Publique, Brazzaville, Republic of the Congo; 7 Department of Anthropology, University of Notre Dame, Notre Dame, 46556 USA; 8 Eck Institute for Global Health, University of Notre Dame, Notre Dame, 46556 USA; 9 Department of Human Behavior, Ecology and Culture, Max Planck Institute for Evolutionary Anthropology, 04103 Leipzig, Germany

**Keywords:** Biological anthropology, Social anthropology

## Abstract

Sleep studies in small-scale subsistence societies have broadened our understanding of cross-cultural sleep patterns, revealing the flexibility of human sleep. We examined sleep biology among BaYaka foragers from the Republic of Congo who move between environmentally similar but socio-ecologically distinct locations to access seasonal resources. We analyzed the sleep–wake patterns of 51 individuals as they resided in a village location (n = 39) and a forest camp (n = 23) (362 nights total). Overall, BaYaka exhibited high sleep fragmentation (50.5) and short total sleep time (5.94 h), suggestive of segmented sleep patterns. Sleep duration did not differ between locations, although poorer sleep quality was exhibited in the village. Linear mixed effect models demonstrated that women’s sleep differed significantly from men’s in the forest, with longer total sleep time (β ± SE =  − 0.22 ± 0.09, confidence interval (CI) = [− 0.4, − 0.03]), and higher sleep quality (efficiency; β ± SE =  − 0.24 ± 0.09, CI = [− 0.42, − 0.05]). These findings may be due to gender-specific social and economic activities. Circadian rhythms were consistent between locations, with women exhibiting stronger circadian stability. We highlight the importance of considering intra-cultural variation in sleep–wake patterns when taking sleep research into the field.

## Introduction

Sleep is a biological process that is inextricably linked to both the physical environment and cultural practices. In order for humans to maintain optimal physiological functioning, sleep and circadian rhythms must synchronize with the physical environment by way of light and temperature entrainment^1,2^. In addition, other exogenous cues such as dietary intake, exercise, and myriad aspects of the social environment also influence sleep–wake regulation^1^. As sleep behavior is closely linked with sociality, it is critical to examine the intersection of these influences on human sleep to understand its cross-cultural expression^3,4^. Much of the existing sleep research has either used sleep surveys from post-industrial societies^5,6^ or has been conducted in laboratory conditions with artificial environmental manipulation^7^. While the latter has established the physiological science of sleep and its importance in health and wellbeing, it provides limited information on how human sleep–wake patterns are situated within an individual’s socio-ecology, including their cultural influences^4,5^. With the advent of actigraphy (non-invasive wristwatches that can record an individual’s 24-h sleep–wake pattern), a biocultural approach can now be implemented in the study of sleep expression in situ across the globe^6,8,9^.

### Subsistence strategies and sleep patterns

Throughout the last decade, sleep studies in small-scale subsistence societies have broadened our understanding of cross-cultural sleep patterns, revealing that human sleep and circadian rhythms are plastic, responding to the environmental and social conditions which individuals inhabit^10–13^. Plasticity refers to the phenotypic adaptation of the circadian system and sleep expression to changing environmental influences that occur in an individual’s lifetime^14,15^. Although the number of studies examining sleep in non-industrial societies remain limited, certain patterns are emerging. For many societies that practice foraging, pastoral and horticultural subsistence, sleep duration is typically shorter than those in post-industrial and urbanized populations^3,5,13^, although there are exceptions^11,16^. Foraging societies in particular display short sleep durations, with averages ranging from 6.23 h in a Hadza community^5^ to 6.97 h in a San community^13^. However, research in small scale subsistence societies has found considerable plasticity in average sleep duration from night to night^3,11,13,17^, a finding that has been attributed to myriad influences, including season, ambient temperature, and behavioral factors^14^. Sleep quality has also been found to be variable in foraging, horticultural, and pastoral populations^5,13,16^. However, small-scale subsistence societies appear to have stronger circadian rhythms (i.e., greater amplitude, less fragmentated and more consistent patterns) that are more entrained to the physical environment as compared to post-industrial societies^3,12,14^. For instance, in a rural, Malagasy population where electricity is not commonplace, sleep duration and efficiency were found to be significantly lower than those of post-industrial populations of Italy and the United States, where light-independent sleep schedules are common^3^. However, circadian rhythms in this Malagasy population were more consistent and stable as compared to post-industrial societies^3^. Similarly, in a study comparing sleep patterns between a rural and an urban community in Mozambique, the rural population exhibited poorer quality sleep but more circadian consistency, while the urban population showed later sleep timing associated with use of electricity in the evening hours^16^. Interestingly, the Mozambique communities showed sleep durations of 8 h on average, irrespective of access to artificial light, suggesting that artificial lighting alone may not affect sleep duration^16^. A trade-off has been hypothesized by Samson, Manus, et al.^3^ to explain these patterns, whereby populations undergoing urbanization gain improved sleep duration and quality through sleep ecologies that are more light, noise, and temperature buffered, but individuals in those settings also lose circadian rhythm entrainment due to the increased use of artificial light.

In societies in which subsistence is largely based on foraging, communities may fission and fuse as they target seasonally available food resources across the landscape^18^. Within the context of these different settlements, variation in daily labor routines as well as housing density and arrangement may impact opportunities for social interaction^19^. Thus, sleep–wake cycles may reflect the specific activities and socio-ecologies of these different locales. To date, sleep patterns for only three societies that subsist at least in part via foraging have been studied: the Hadza of Tanzania^5,13^, the Ju/’hoansi San of Namibia^13^, and the Tsimane of Bolivia^13^. However, these prior studies did not compare sleep and circadian rhythmicity between different seasonal settlements, which may elucidate the flexibility of sleep patterns in response to varying social and subsistence activities. To this end, our paper aims to examine sleep biology in a foraging BaYaka community from the Republic of Congo who move fluidly between a denser village setting and smaller, forest camps as they access seasonal resources.

BaYaka describe the forest as calm, cool, and peaceful, and the village as hot (climatically and socially), noisy and dangerous^20,21^. The use of artificial light is minimal in both locations. Increased population density, however, is associated with higher noise levels and increased opportunities for nighttime socialization, which can reduce sleep quality and alter circadian rhythms^3^. There are also differences in the types of domiciles constructed in the village versus the forest. Compared to the mudbrick homes used in the village, BaYaka sleep in leaf huts in the forest, which may increase exposure to circadian entrainment cues, most especially temperature and light. We highlight the importance of considering intra-cultural variations in sleep–wake patterns, which may be linked to location-based sleep differences.

### Gender-based differences in sleep ecology

Despite the biological necessity of sleep, individuals balance their sleep time with social and subsistence demands^22^. Humans incur sleep debt for increased opportunities of socialization, information gathering, caregiving, and work activities^17,23,24^. These activities are often rooted in social role expectations between men and women, leading to gender-differentiated sleep outcomes. For example, Maume and colleagues^25^, using data from the 2006 European Social Survey, found that having a child under 6 in the home significantly increased the odds of a woman sleeping restlessly, whereas it was not linked to men’s sleep. This association may be different in cultural contexts in which both shared family sleep environments and some nighttime care by fathers is common.

Co-sleeping both with children and other adults is a valued cultural practice in many societies and is also often a necessity for households in which indoor sleeping space is limited, as is common among mobile foragers^6,26^. Among the Hadza of Tanzania, actigraphic analysis revealed that co-sleeping with a breastfeeding infant was not linked to altered sleep duration in mothers, but a higher number of co-sleepers was correlated with shorter sleep duration and quality^27^. However, breastfeeding was associated with earlier wake times^27^, which is consistent with findings from the United States^28,29^. Relatively little is known about fathers’ nighttime caregiving^30^, but shared family sleep has been linked to lower testosterone levels in cosleeping fathers in the Gambia and the Philippines^31,32^. Additionally, and relevant to the present paper, BaYaka fathers are regularly involved in hands-on care of infants and young children^20,33^.

Beyond co-sleeping, gendered work and divisions of labor between men and women may also affect sleep, but this is little explored outside of industrial and post-industrial contexts. In all forager societies, men and women practice some specialization in different subsistence activities, with men often engaging in more hunting and women typically gathering more intensely, though considerable variation in these roles has been reported^34^. However, in Hadza adults, Samson et al.^5^ found no overall difference in sleep patterns between men and women. Similarly, Yetish et al.^13^ reported no difference in sleep duration in the Tsimane, though men exhibited more variability in sleep onset^17^. This may be linked to collaboration in subsistence activities. For example, in the BaYaka community surveyed here, Sarma et al.^35^ found important complementarities in men and women’s work—BaYaka men and women will work side-by-side in gardens, with men clearing plots and women planting. Unlike other societies where heightened gender distinctions in types and intensities of subsistence roles drive significant differences in day and nighttime activity between men and women^36^, it is possible that BaYaka sleep patterns are similar between men and women because of a less rigid gendered division of labor.

Here, we studied the sleep patterns of BaYaka foragers (n = 51) from the Republic of Congo. The primary objectives of this study were to (1) characterize the overall sleep patterns of BaYaka adults in this community (2) examine whether there were any differences in these sleep and circadian variables in the same community between the village and forest locations, and (3) investigate whether gender-based differences occurred in sleep duration, sleep quality, sleep timing, and circadian rhythmicity. We hypothesized that sleep and circadian rhythms would differ between the forest and the village. Specifically, we predicted that BaYaka study participants would exhibit poorer sleep quality, shorter sleep duration, later sleep onset (i.e., the time that participants fell asleep), later sleep end (the time they woke), and less robust circadian rhythms (more fragmentation and less stability in rhythm) when residing in the village as compared to the forest. With respect to gender, we hypothesized that we would observe little difference between men and women in either circadian or sleep variables. Specifically, we predicted that sleep duration, sleep quality, and sleep onset and end would not vary significantly between men and women. Likewise, we predicted that circadian rhythms for both men and women would show similar entrainment to their environment.

## Results

Descriptive sleep characteristics and Non-Parametric Circadian Rhythm Analysis (NPCRA) for this sample are presented in Table 1. The average Total Sleep Time (TST) was 5.94 h (standard deviation = 1.45), with Twenty-four hour Total Sleep Time (TTST) reported at 6.95 h (SD = 1.82). Sleep efficiency was 66.4% (SD = 11.32). Time in Bed (TIB) was 8.95 h (SD = 1.67). The average nap period duration was 1.01 h (SD = 1.10). On average, sleep onset occurred at 21:05 (SD = 1:34) while sleep end occurred at 05:42 (SD = 1:25). Descriptive statistics for sleep variables between the two locations (village and forest) demonstrate differences in sleep quality (Table 2). Sleep values were similar between locations, apart from a higher probability of increased sleep efficiency in the forest (village = 65.1%, forest = 68.6%, Bayes Factor (BF) = 6.69) as well as decreased sleep fragmentation (village = 52.52, forest = 47.07, BF = 5.15). NPCRA analysis revealed that the BaYaka exhibited relatively consistent circadian rhythms between locations, with an overall interdaily stability of 0.55, intradaily variability of 0.11, and a relative amplitude of 0.86. While NPCRA analysis yielded weak evidence for a difference in intradaily variability between the forest and village locations (BF = 2.55), no other support for a difference in circadian rhythms was found.

**Table 1:** Descriptive sleep quotas (n = 51, men = 23) and NPCRA analysis (n = 31, men = 17) of the BaYaka from actigraphic analysis.

**Sleep quota**	
Sleep onset	21:05 (1:34)
Sleep end	5:42 (1:25)
Time in bed (h)	8.95 (1.67)
Total sleep time (h)	5.94 (1.45)
Twenty-four hour total sleep time (h)	6.95 (1.82)
Nap period duration (h)	1.05 (1.10)
Wake after sleep onset (h)	2.53 (1.10)
Sleep efficiency (%)	66.4 (11.32)
Sleep fragmentation	50.47 (18.04)
**NPCRA values**	
Relative amplitude	0.86 (0.08)
Interdaily stability	0.55 (0.11)
Intradaily variability	0.11 (0.06)
L5	2230 (1550)
M10	32,019 (11,381)
M10 Onset	7:27 (0:34)

Overall age range is 17–72 years. Data are reported as mean (standard deviation). Nap period duration was calculated by subtracting TST from TTST. L5 is the activity during the 5 h of least activity during the diel cycle. M10 is the activity during the most active 10 h during the diel cycle.

**Table 2:** Study sample characteristics by village (n = 39 individuals) and forest (n = 23 individuals), including sleep quotas and NPCRA analysis (village = 16 individuals, forest = 15 individuals).

	Village	Forest	Bayes factor or t-test	BF interpretation
**Sleep quota**				
Sleep onset	21:08 (1:28)	21:02 (1:42)	t = 0.55, *p* = 0.58	
Sleep end	5:34 (0:41)	5:56 (2:07)	t = -1.97, *p* = 0.05	
Time in bed (h)	8.95 (1.51)	8.93 (1.90)	0.12	Modest evidence for H0
Total sleep time (h)	5.84 (1.47)	6.10 (1.41)	0.44	Weak evidence for H0
Twenty-four hour total sleep time (h)	6.83 (1.69)	7.15 (2.01)	0.41	Weak evidence for H0
Nap period duration (h)	0.99 (1.01)	1.05 (1.24)	0.13	Modest evidence for H0
Wake after sleep onset (h)	2.59 (1.09)	2.44 (1.12)	0.24	Modest evidence for H0
Sleep efficiency (%)	65.08 (12.08)	68.61 (9.58)	6.69	Modest evidence for H1
Sleep fragmentation	52.52 (19.66)	47.07 (14.40)	5.15	Modest evidence for H1
**Non-parametric circadian rhythm analysis**				
Relative amplitude	0.87 (0.04)	0.86 (0.11)	0.34	Weak evidence for H0
Interdaily stability	0.55 (0.10)	0.55 (0.12)	0.34	Weak evidence for H0
Intradaily variability	0.09 (0.05)	0.135 (0.05)	2.55	Weak evidence for H1
L5	2096.56 (675.47)	2372.20 (2149.57)	0.37	Weak evidence for H0
M10	30,011.56 (7672.68)	34,160.13 (14,315.79)	0.50	Weak evidence for H0
M10 onset	7:30 (0:38)	7:24 (0:37)	t = 0.49, *p* = 0.63	

Data are presented as mean (standard deviation). Bayes factor is reported to assess differences in sleep quotas between the locations. Time value differences are assessed using t-tests.

To test gender-based sleep differences*,* we examined men and women's sleep between the village and forest locations. While there were no differences for men’s sleep variables between the village and forest (all Bayes factors favored the null hypothesis), women’s sleep substantially differed between the two sites. A strong association for differences in sleep duration and quality between locations was found; women in the forest had a greater probability of longer total sleep time compared to women in the village (village = 5.91 h, forest = 6.53 h; BF = 10.27), as well as longer TTST (village = 6.60 h, forest = 7.43 h; BF = 18.92). In addition, there was extremely strong evidence for higher sleep efficiency in the forest (village = 65.25%, forest = 71.43%; BF = 161.29), and strong evidence for lower sleep fragmentation (village = 53.28, forest = 45.58; BF = 8.72). This suggests more consolidated sleep patterns for women when they reside in the forest location as compared to the village.

When comparing gender-based sleep differences *within* each location (see Table 3), no differences were found between men and women in the village location, with the exception of weak evidence for longer total sleep time in women (women = 5.91 h, men = 5.77 h; BF = 1.03). However, Bayes factors indicated very strong evidence for women exhibiting increased sleep efficiency in the forest camp in comparison to men’s sleep (71.4% vs. 65.6%; BF = 76.2), as well as increased total sleep time in the forest compared to men (6.53 h vs. 5.65 h; BF = 128.1) (Table 4). Using a F-test of variance, we found that men exhibited greater variance in sleep onset in the forest location compared to women (mean = 20:47, variance = 0:05; men’s mean = 21:17, variance = 0:09; *p* = 0.004), but not in the village location (*p* = 0.24). In addition, men exhibited greater variance in sleep end (i.e., time they wake) in the forest location compared to women (mean = 5:43, variance = 0:01; men’s mean = 6:09, variance = 0:22, *p* < 0.001). In other words, in the village, men go to bed and wake around the same time as the women. In the forest, however, men’s sleep timing (both sleep onset and sleep end) is highly variable compared to women's sleep timing.

**Table 3:** Comparison of sleep variables between the village (women, 110 nights of data vs. men, 116 nights) and forest (women, 70 nights vs. men, 66 nights) sites.

	Village, women	Village, men	Bayes factor or t-test	Forest, women	Forest, men	Bayes factor or t-test
**Sleep quotas**						
Sleep onset	20:57 (1:25)	21:18 (1:31)	t = − 1.83, *p* = 0.07	20:47 (1:24)	21:17 (1:57)	t = − 1.73, *p* = 0.09
Sleep end	5:32 (0:28)	5:35 (0:50)	t = − 0.6, *p* = 0.54	5:43 (0:29)	6:09 (3:00)	t = − 1.16, *p* = 0.25
Time in bed (h)	9.0 (1.37)	8.91 (1.63)	0.18	9.17 (1.46)	8.68 (2.26)	0.52
Total sleep time (h)	5.91 (1.47)	5.77 (1.47)	1.03	6.53 (1.10)	5.65 (1.55)	128.1
Twenty-four hour total sleep time (h)	6.60 (1.63)	7.06 (1.72)	0.15	7.43 (1.76)	6.85 (2.21)	0.68
Nap period duration (h)	0.69 (0.95)	1.28 (0.99)	2521	0.90 (1.22)	1.21 (1.25)	0.48
Wake after sleep onset (h)	2.67 (0.98)	2.51 (1.17)	0.17	2.40 (0.86)	2.49 (1.34)	0.20
Sleep efficiency (%)	65.24 (11.94)	64.92 (12.26)	0.26	71.43 (7.21)	65.62 (10.85)	76.2
Sleep fragmentation	53.28 (19.11)	51.79 (20.22)	0.85	45.58 (13.30)	48.64 (15.43)	0.37

Results are reported as mean (standard deviation). Bayes factor is reported to assess differences between sleep quotas. Time value differences (sleep onset, sleep end) are assessed using t-tests.

**Table 4:** Descriptive sleep quotas (n = 51, men = 23) and NPCRA analysis (n = 31, men = 17) comparing women and men in the BaYaka study sample.

	Women	Men	Bayes factor or t-test	BF interpretation
**Sleep quotas**				
Sleep onset	20:53 (1:24)	21:18 (1:41)	t = − 2.54, *p* = 0.01	
Sleep end	5:36 (0:29)	5:47 (1:56)	t = − 1.27, *p* = 0.21	
Time in bed (h)	9.06 (1.40)	8.83 (1.88)	0.28	Weak evidence for H0
Total sleep time (h)	6.15 (1.37)	5.73 (1.50)	5.31	Modest evidence for H1
Twenty-four hour total sleep time (h)	6.92 (1.73)	6.98 (1.91)	0.41	Weak evidence for H0
Nap period duration (h)	0.77 (1.06)	1.26 (1.09)	731.2	Very strong evidence for H1
Wake after sleep onset (h)	2.57 (0.94)	2.50 (1.23)	0.13	Weak evidence for H0
Sleep efficiency (%)	67.64 (10.77)	65.18 (11.70)	0.93	Weak evidence for H0
Sleep fragmentation	50.28 (17.46)	50.65 (18.64)	0.12	Weak evidence for H0
**Non-parametric circadian rhythm analysis**				
Relative amplitude	0.90 (0.04)	0.83 (0.10)	3.02	Modest evidence for H1
Interdaily stability	0.59 (0.10)	0.51 (0.11)	1.66	Weak evidence for H1
Intradaily variability	0.08 (0.04)	0.14 (0.05)	30.93	Very strong evidence for H1
L5	1854.78 (525.82)	2538.88 (2014.54)	0.61	Weak evidence for H0
M10	38,673.07 (13,420.28)	26,539.06 (5,085.21)	19.76	Strong evidence for H1
M10 onset	7:42 (0:28)	7:14 (0:34)	t = 2.59, *p* = 0.01	

Data are reported as mean (standard deviation). Bayes factor is reported to assess differences in sleep quotas between genders. Time value difference (sleep onset, sleep end, and M10 onset) is assessed using a t-test.

NPCRA analysis between men and women (see Table 4) showed strong evidence that women had an increased probability of lower intradaily variability (women = 0.08, men = 0.14; BF = 30.93) as well as higher activity levels (Most active consecutive 10-h values, or M10) compared to men (women = 38,673, men = 26,539; BF = 19.76). However, the onset of men’s M10 hours was significantly earlier compared to women (women = 07:42, men = 07:14, *p* = 0.01). There were also slight differences in relative amplitude (BF = 3.02) and interdaily stability (BF = 1.66) between men and women.

**Figure 1:**
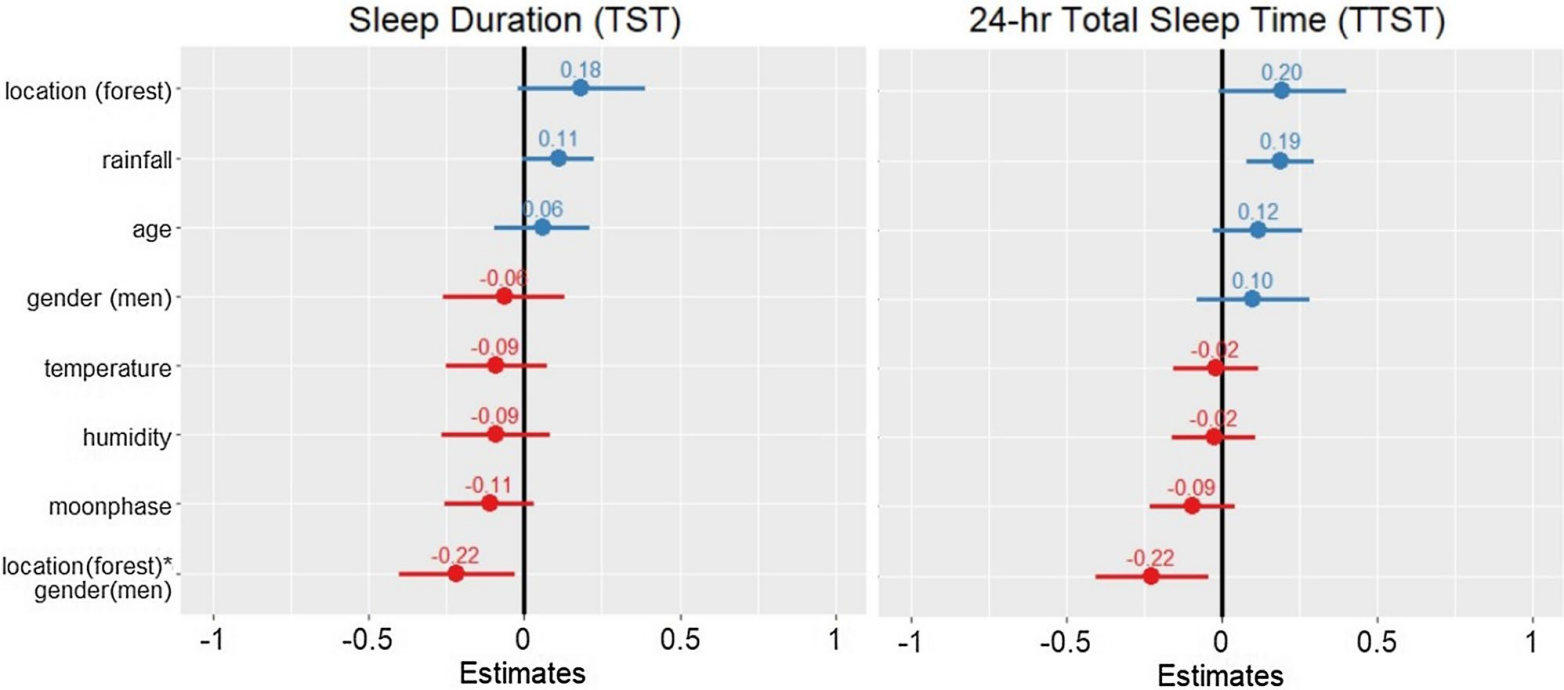
Two fixed effect plots of ecological predictors on sleep duration quotas: nighttime sleep duration (TST) (left) and 24-h total sleep time (TTST) (right). Reference variables are set as *village* for location, and *women* for gender for all models. Both plots illustrate positive (indicated in blue) and negative (indicated in red) predictors of TST and TTST. The plotted lines indicate 95% confidence intervals of each predictor variable. For TST, only the location * gender interaction was a significant predictor variable. The location * gender interaction was also a negative predictor of TTST, while rainfall was a positive predictor. Values written above each plotted line are standard estimates of each predictor. Continuous predictors were scaled for comparability of the coefficients in the models.

**Figure 2:**
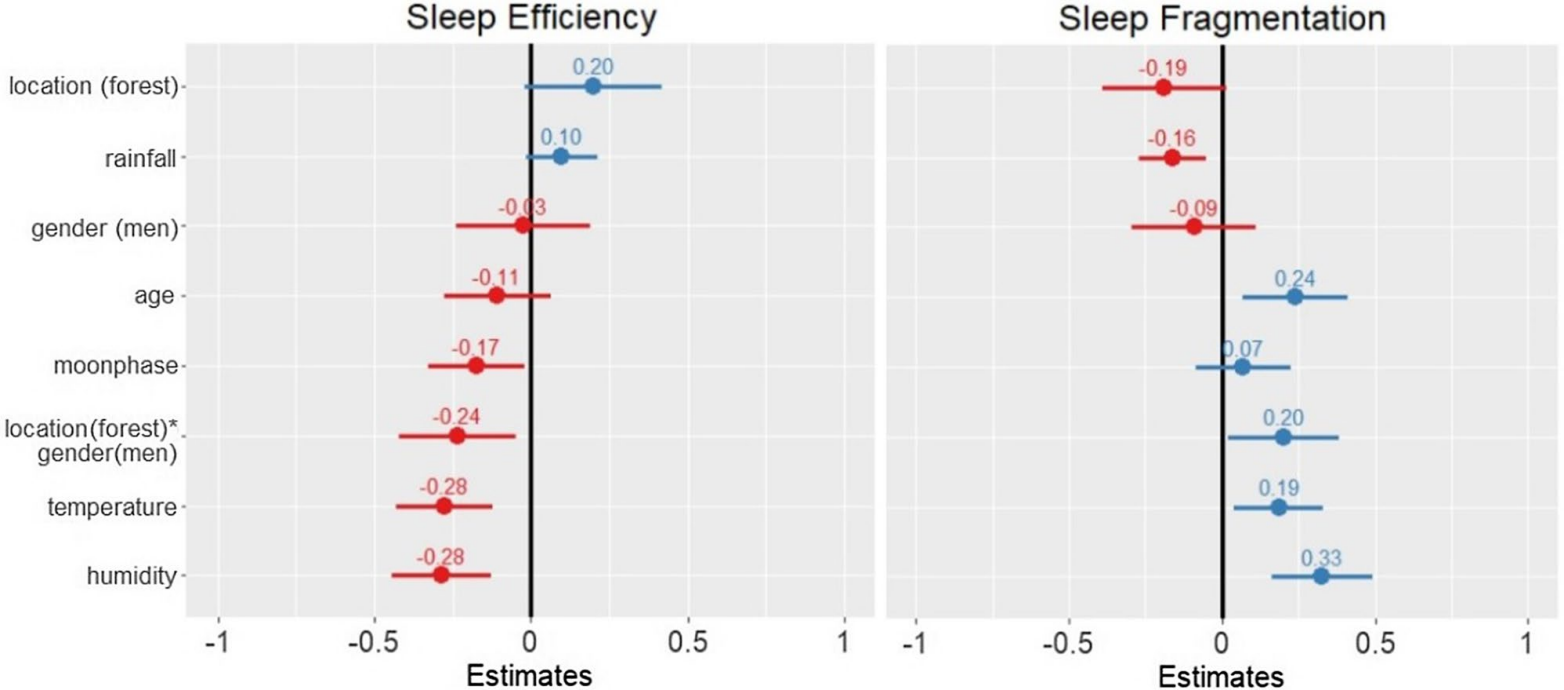
Two fixed effect plots of ecological predictors on sleep quality measures: sleep efficiency (left) and Sleep fragmentation (right). For both plots, positive predictors on sleep quality are indicated in blue, while negative predictors are indicated in red. Values written above each confidence line are standard estimates of each variable. The plot on the left illustrates that humidity, temperature, moon phase, and the location * gender interaction effect all negatively influenced sleep efficiency in the BaYaka. The plot on the right illustrates that rainfall negatively influenced sleep fragmentation, while humidity, temperature, age, and the location * gender interaction effect were positive predictors of sleep fragmentation.

Linear mixed effects models also revealed significant associations between sleep and gender contingent upon location. With references set as *women* for gender and *village* for location for all models, an interaction between gender * location was negatively associated with TST (β ± SE =  − 0.22 ± 0.09, 95% confidence interval (CI) = [− 0.4, − 0.03]), as well as TTST (β ± SE =  − 0.22 ± 0.09, CI = [− 0.4, − 0.04]), indicating a difference in TST and TTST between men and women in the forest but not village locations (see Fig. 1). Additionally, sleep efficiency was significantly associated with the gender * location interaction (β ± SE =  − 0.24 ± 0.09, CI = [− 0.42, − 0.05]), whereas sleep fragmentation was significantly positively associated with the interaction (β ± SE = 0.20 ± 0.09, CI = [0.02, 0.38]), indicating that men’s sleep was less efficient and more fragmented in the forest as compared to women (see Fig. 2).

In the study location, the average time of sunrise in July and August was 05:47, while sunset occurred at 17:58. During these months, average nighttime temperatures ranged from 21 to 25 °C, with an average humidity of 84%. Examining sleep quality in relation to environmental factors, linear mixed effects models demonstrated that sleep efficiency significantly decreased with temperature (β ± SE =  − 0.28 ± 0.08, CI = [− 0.43, − 0.12]) and humidity (β ± SE =  − 0.28 ± 0.08, CI = [− 0.44, − 0.125]) (see Fig. 2). Rainfall was a positive predictor of TTST (β ± SE = 0.19 ± 0.05, CI = [0.08, 0.30]) (Fig. 2). Humidity, temperature, and age were positive predictors of sleep fragmentation (humidity: β ± SE = 0.33 ± 0.08, CI = [0.16, 0.49]; temperature: β ± SE = 0.19 ± 0.07, CI = [0.04, 0.33]; age: β ± SE = 0.24 ± 0.09, CI = [0.07, 0.41]) (Fig. 2). In addition, sleep fragmentation significantly decreased with rainfall (β ± SE =  − 0.16 ± 0.06, CI = [− 0.27, − 0.05]).

**Figure 3:**
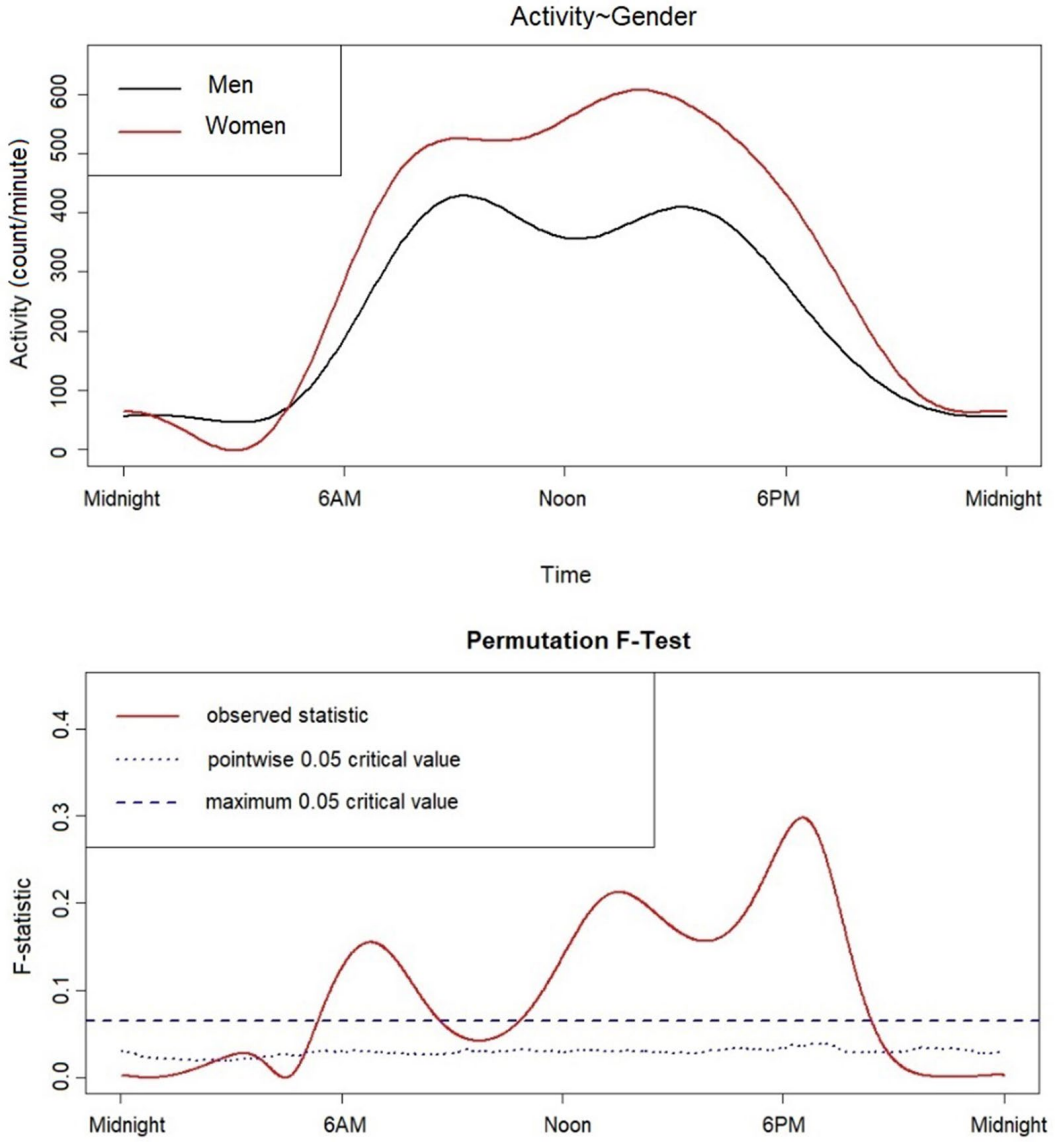
Functional linear modelling comparing 24-h sleep–wake patterns of BaYaka men and women. Women are represented by a red line and men represented by a black line. Women displayed a sharp, steady incline of activity in the morning and generally higher levels of mean activity throughout the day as compared to men, especially in the afternoon. Men show a decline in their activity towards noon, suggestive of resting and/or napping periods before the next peak of activity, and a steady decline throughout the evening. The bottom panel displays a permutation F test; the point-wise critical value (dotted line) displays the proportion of permutation F values at the significance level of 0.05 for every time point. Men and women have significantly different mean circadian activity patterns at the points when the observed F-statistic (represented as a solid line) crosses the critical value (dotted line).

**Figure 4:**
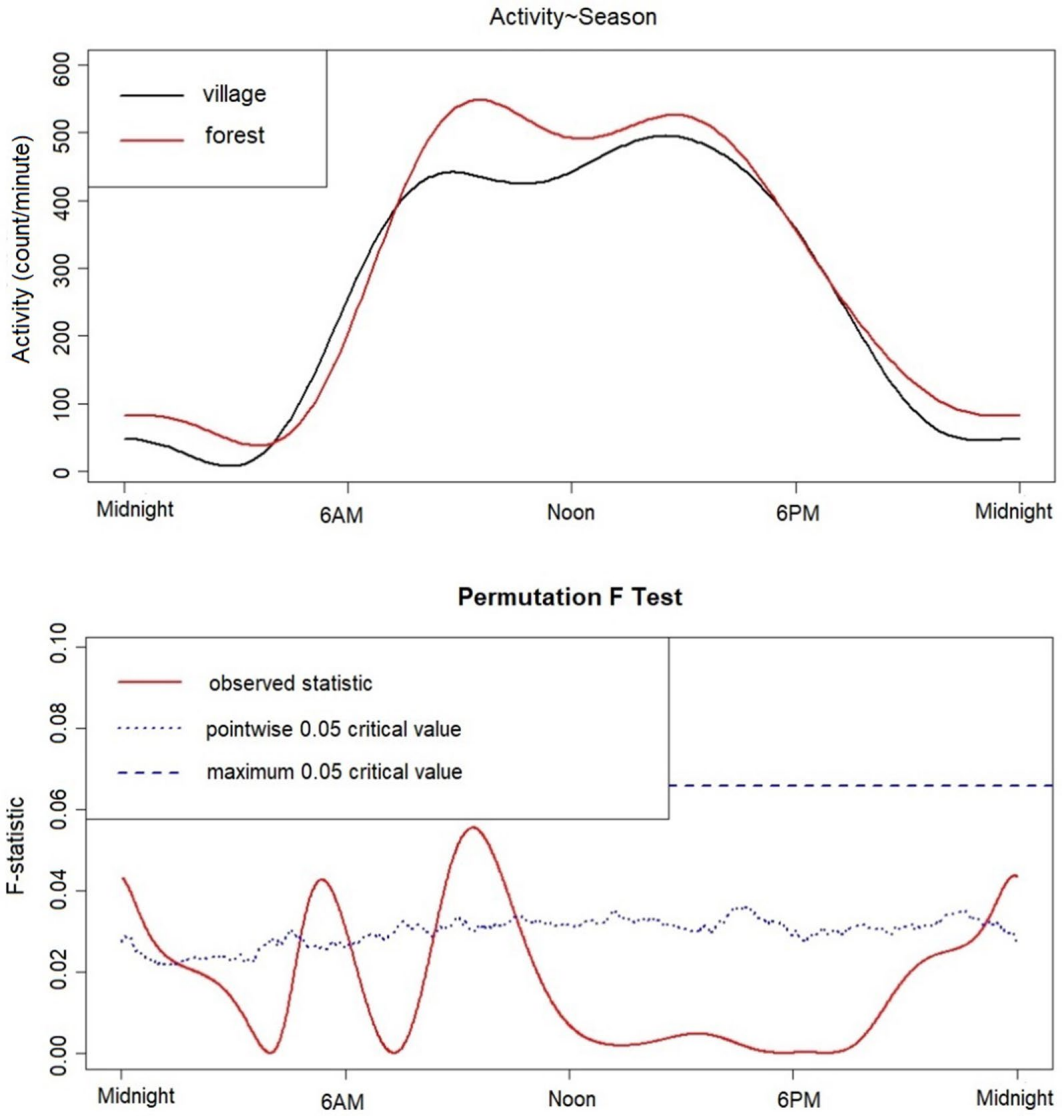
Functional linear modelling comparing 24-h sleep–wake patterns of BaYaka in the forest and village locations. The forest location is represented by a red line and the village represented by a black line. Bottom part of the illustration displays the permutation F test; significant values of the F-statistic cross the line of the critical value (dotted line).

We used functional linear modelling to demonstrate 24-h circadian activity patterns between men and women overall (see Fig. 3). Women exhibited significantly higher activity levels compared to men throughout most of the day. Figure 3 suggests that both men and women displayed a first peak of high-level activity in the mid-morning; however, men’s activity declined mid-morning and increased again just after noon (though to a lesser extent than their first peak), whereas women’s activity levels plateaued in the mid-morning and declined slightly before increasing again to an even greater activity level in the afternoon. Women also exhibited lower activity levels around ~ 3:00 a.m. and a sharper incline of activity in the morning after waking (~ 6:00 a.m.) compared to men. Functional linear modelling of the forest and village locations (with no differentiation between men and women) was also conducted (Fig. 4). Results illustrated a significant difference between the locations in the evening hours, with more activity around midnight hours noted in the forest location as compared to the village. The forest location also displayed higher activity levels from ~ 9:00 a.m. until noon. Both locations displayed a similar pattern of a decrease in activity from mid- to late morning (although to a greater extent in the forest), followed by a short rebound of activity in the afternoon before the steady evening decline. While peak activity in the village occurred in the afternoon, the greatest activity in the forest occurred in the morning.

## Discussion

This study examined sleep and circadian rhythms in a BaYaka community that resides in and moves between two socio-ecologically distinct locations. Our findings indicate that BaYaka sleep and circadian rhythmicity are highly entrained to the physical environment and remain consistent even with changes in location. We found moderate support for the hypothesis that sleep is of poorer quality in the village compared to the forest location. Additionally, our hypothesis that sleep would be relatively similar between men and women was only partially supported. While sleep variables were similar in the village location, we found significant gender-based sleep differences in the forest location, pointing to the potential influence of gender-specific social and labor activities on sleep patterns within the same population.

Our prediction that BaYaka would exhibit stronger circadian rhythmicity in the forest location was not supported—NPCRA analysis revealed consistent circadian rhythms irrespective of location, with no differences in relative amplitude (rhythm strength) and interdaily stability (rhythm consistency), and only a slight difference in intradaily variability (rhythm fragmentation). While sunlight and temperature are relatively similar in both locations, the differences in built environment between the forest and village were predicted to exert a strong enough effect to alter circadian rhythms between locations. However, differences in location-specific domicile construction did not appear to alter circadian rhythms, likely because this community continues to spend much of their waking time outside. Our findings for stable circadian rhythms in this community aligns with the circadian entrainment hypothesis proposed by Samson, Manus and colleagues^3^, where high levels of exposure to the physical environment (in particular, sunlight and outdoor temperature) during waking hours leads to greater levels of circadian stability and uniformity as compared to post-industrial populations that largely use artificial lighting and temperature control.

There was moderate support for our prediction that sleep would be adversely affected by being in the village location. BaYaka exhibited lower sleep efficiency in the village (65.1%) compared to the forest (68.6%), with higher sleep fragmentation in the village. Based on ethnographic observation, we believe this may be due to the higher population density, greater noise, and more social interaction within the village. Interestingly, sleep duration did not substantially differ between the two locations, nor did average sleep time (both onset and end). BaYaka sleep end corresponded roughly to dawn; early wake times have been described in populations exposed to high levels of natural light throughout the day, which synchronizes their circadian clock with solar time^2^. In particular, a morning dose of bright sunlight is considered the most potent circadian entrainment factor for sleep timing^1^. Sleep onset occurred approximately 3 h after sunset, corresponding with use of fires in the evening hours to increase work and social opportunities—echoing findings of populations in similar subsistence settings by Yetish and colleagues^13^ among San peoples, and Beale and colleagues^16^ working in a rural community in Mozambique.

Associations between sleep quality and environmental conditions were found at both locations. Nocturnal temperature and humidity exerted a significant negative influence on sleep efficiency, with higher sleep fragmentation. As BaYaka domiciles in this community do not have doors, thermal stress could be reducing sleep quality—a similar finding to that of Samson, Crittenden and colleagues in their study with Hadza^5^. Total sleep time did not appear to be altered by any of the environmental factors examined; however, periods of increased rainfall influenced both TTST and sleep fragmentation, suggesting an increase in napping during rainy days, as people remain in-doors and tend to engage in fewer activities. Forest camps are in walking distance of the village, and thus it is unlikely that temperature, rainfall and humidity significantly differ between the two locations; however, future research that monitors specific environmental conditions within each location may provide more detail on the influence of environmental conditions on sleep behavior in this community.

We found mixed results for our hypothesis that BaYaka would exhibit minimal gender-based differences in sleep quotas or circadian rhythms. No strong differences in sleep variables between men and women were found in the village location, but in the forest camp, women exhibited longer total sleep time and higher sleep efficiency than men. Overall, women also exhibited less circadian fragmentation than men, and slightly stronger and more stable circadian rhythms. It is possible that, despite labor coordination between BaYaka men and women^20,35^, gender-differentiated work and social activities, particularly in the forest location, may drive gender differences in sleep expression and in circadian rhythms. Functional linear modelling analysis revealed that women had significantly higher activity levels than men, with a sharp rise in activity almost immediately after waking, which remained higher than men for the remainder of the day. Men’s activity levels, comparatively, were lower than women’s, with a slower activity increase in the morning. This slower slope in men’s morning activity is potentially due to the higher activity period for men throughout the night; men participate in nighttime socialization, hunting and fishing, which may be skewing the finding of an earlier Most Active 10 h (M10) in men (07:15) compared to women (07:42).

Conversely, women exhibited longer, higher quality sleep throughout the night in the forest location. This is surprising, given that a total of 5 women in the forest were breastfeeding and cosleeping with infants under two years old, which has been linked to more frequent nighttime arousals in mother-infant dyads in the global North^22,26,37^. The proportion of breastfeeding women (42% of women in the village, and 38% of women in the forest) are roughly equivalent, and thus likely do not explain the locational gender-based differences in sleep duration and quality. Sarma and colleagues^35^ reported that BaYaka women in the village engaged for longer periods in moderate to vigorous subsistence activities compared with men. While BaYaka women work consistently over longer periods of the day, men participate in inconsistent spurts of high energy activity, such as hunting large game or climbing trees^35^. In the forest camps, where energetics of the BaYaka have not yet been reported, it may be that gender-based differentiation of forest hunting and foraging tasks drives the gender-differentiated sleep patterns observed in this study. While our study did not find differences between men and women’s sleep in the village (apart from a slight difference in total sleep time), women’s work activities in the forest may substantially differ from that of the village, creating stronger labor demands and thereby affecting sleep behavior. While women maintain gardens and continue to forage when living in the village, the forest portion of our study was conducted during caterpillar and bail-fishing seasons, leading to a potential increase in the intensity and duration of women’s work. Specifically, women travel throughout the day between caterpillar trees, and, during bail-fishing, perform the majority of the digging as they change the course of streambeds to isolate fish for capture.

Activity patterns related to social space appear to have affected sleep timing variability. BaYaka camps are small and close-knit, particularly in the forest location. This social density may increase the likelihood that the nighttime activities of some individuals disrupt or fragment the sleep of others, or individuals may choose to join an activity if it is of interest to them. BaYaka women had more consistent wake and bedtimes in this study; this aligns with findings from studies in post-industrial societies^38^, and can be attributed to morning household responsibilities and childcare. Conversely, men displayed a high variance in both sleep onset and end, with an especially high standard deviation in sleep end in the forest camp. This is in contrast to the Tsimane horticulturalists of Bolivia, where sleep end was relatively stable from day-to-day^17^. Thus, the social influence on sleep timing may also influence circadian rhythm expression—the high variability in sleep timing in men may contribute to their higher circadian fragmentation and lower stability of circadian rhythms compared to women. BaYaka men opportunistically hunt and fish at night providing there is sufficient natural or artificial illumination—potentially reflecting the negative association between sleep efficiency and moon phase that we have documented elsewhere^39^. Our finding of high levels of sleep variability in men but not in women aligns with the hypothesis posited by Maume et al.^22^, whereby women in post-industrial societies often trade-off socialization opportunities for increased sleep time, while leisure and social time is often more incorporated into men’s evening activities. Ethnographic observations confirm that BaYaka men preferentially choose to socialize at night in preparation for hunting or fishing, particularly in the forest location. In addition, men’s socialization in the village location often involves alcohol ingestion; while this has demonstrated effects on sleep quality^40,41^, this was not demonstrated in our study, as no difference in sleep variables between men and women in the village location was found.

Overall, the BaYaka community in this study exhibited similar sleep patterns to Hadza^5^, Tsimane^13^, and San^13^ communities, adding to and complementing the limited knowledge of sleep patterns in subsistence foraging societies. Nighttime sleep duration in all four populations averaged less than 7 h, notably less than the 8 h of monophasic sleep typically recommended by clinicians in Euro-American post-industrial populations^42^. Like Hadza^5^, low sleep duration among BaYaka occurs despite a high amount of time in bed during the night (8.95 h on average).

The high level of sleep timing variability in this BaYaka community, combined with high levels of sleep fragmentation, and longer 24-h total sleep time compared to nighttime sleep duration is suggestive of segmented sleep patterns. While a single, consolidated sleep bout is considered the healthy standard in post-industrial nations^42^, segmented sleep patterns were common in both European and equatorial populations prior to the widespread use of electricity in the twentieth century^43^. Plasticity in sleep and circadian expression is an essential feature of adaptation to different ecologies, and has been found to be more pronounced in urbanized populations exposed to electric light^15^. Our study suggests that subsistence and social factors may also drive high sleep flexibility, even when daylength is relatively stable and access to electrical light is minimal. The finding of sleep/wake variability, particularly between locations, in association with consistent circadian rhythms may have implications for sleep and health disparities. In post-industrial society, negative health consequences reported with short sleep duration and high sleep variability are often exacerbated by circadian misalignment, increasing inflammation and diabetes risk^24,44^. To date, no study has examined the influence of sleep and circadian variables on health outcomes in foraging societies. Future research into associations between sleep timing variability, circadian rhythms, and health outcomes in small-scale subsistence societies could explore this possibility. For example, it is possible that circadian rhythms that are more closely aligned with the physical environment may be somewhat protective against the negative health consequences of poor sleep.

In addition, future research that incorporates co-sleeping (particularly the number of co-sleepers), as well as variables such as nursing and pregnancy could elucidate further gender-based differences in the sleep behaviors of BaYaka adults. Interestingly, our study had two noted differences in comparison to the studies of the San and the Hadza. Our finding of gender-based sleep differences occurring within different residential locations has not been previously described in forager societies and highlights the importance of an understanding of sleep behaviors in varying contexts within a single community. In addition, circadian rhythms in this BaYaka community were stable and consistent, even more so than the circadian rhythms studied in the Hadza^5^. The intradaily variability of circadian rhythms in the BaYaka (0.11) is the lowest circadian fragmentation reported in small-scale, subsistence populations thus far^3, 12^, and highlights the wide physiological variability in circadian rhythmicity, and the flexibility of these rhythms in response to social and ecological factors.

## Conclusion

We examined sleep and circadian rhythms among BaYaka foragers inhabiting small forest camps and a relatively more populous village settlement—settings that are in relatively close geographical proximity and thus share environmental conditions, but socio-ecologically differ. Our results demonstrated gender differences in sleep behavior that we argue may be due to differences in subsistence activities and social opportunities between the two locations. Our findings add to our understanding of sleep in populations who are minimally exposed to artificial light and controlled, uniform temperatures. Like other subsistence societies, BaYaka participants showed stable circadian rhythms, short sleep durations, and relatively low sleep efficiency. Cross-cultural sleep research grounded in socioecological frameworks is needed to illuminate not only the plasticity of human sleep and its adaptive functions, but to provide further context for understanding both sleep needs and sleep disorders across the globe^4,45^. Our investigation of sleep among BaYaka adds to this knowledge by revealing how certain sleep profiles may be expressed in the same population under different conditions. In turn, understanding the relationship between socio-ecology and sleep patterns adds to the cross-cultural exploration of sleep, and provides insight into human variation of sleep and circadian biology.

## Methods

### Description of study site

BaYaka who participated in the present study are mobile forest foragers who spend half the year in a multi-ethnic village setting, and the other half living in small forest camps^46^. Forest camps have a population density of 7–43 individuals with the mean area per person averaging 11.5 m^2,47^. These camps are typically oriented around a core group of siblings, their spouses, children, and elders. In contrast, in the village these dispersed camps coalesce into a larger community of ~ 200 people situated in an ethnically-segregated neighborhood adjacent to those of Bondongo fisher–farmers who occupy the village year round^46^. In both settings, BaYaka primarily rely on forest resources such as wild yams (e.g. *Dioscoera* sps.), edible leaves, mushrooms, fish, and wild game. Many BaYaka also keep swidden garden plots in the forest near the village where they grow plantains and manioc^46^. In the village they also trade forest products or labor for garden produce to the Bondongo for market goods (e.g. salt, cigarettes, Maggi bouillon cubes), alcohol or, occasionally, cash. Within the domain of subsistence, BaYaka maintain a gender-based division of labor^20,34,35^, although gender roles are conceived of as complementary^48,49^. Men hunt and climb trees to collect palm nuts and palm wine, whereas women perform bail fishing, and do a majority of gathering and food preparation tasks. In general, these gendered subsistence roles are collaborative and can be flexible^20,35^. Men and women will cooperate in barrier fishing and garden work, for instance. Additionally, BaYaka men provide hands-on infant and childcare^20^.

### Data collection

Data collection occurred in two field seasons. In the village, data was collected in July through September 2017. Data from the forest camp was collected in July through September 2018. Overall, 39 participants took part in the village portion of the study (men = 20), and 23 individuals took part in the forest portion of the study (men = 10). A subset of individuals (n = 11; men = 7) participated in both the forest and village data collection. Throughout the study period, 11 women had a baby under 2 years who breastfed and co-slept with the mother (village = 8, forest = 5; 2 women participated in both field seasons). As BaYaka do not record their age in years, age data was estimated following methods by Diekmann et al.^50^. Ages in this study ranged from 17 to 72 years (mean = 36, standard deviation = 13.75). Participants wore actigraphy watches for 2 weeks each season. A total of 362 nights of sleep data was analyzed in this study (village = 226 nights, forest = 136 nights, range = 3–8 days per participant).

Average daily temperature, humidity, and rainfall data, as well as sunrise and sunset times, were retrieved from WorldWeatherOnline.com (2019) for each sleep night, from a weather station in Impfondo, Republic of Congo. We averaged the data for temperature and humidity four times each night (at 21:00, 0:00, 03:00, and 06:00) to obtain mean nightly averages for each day of the data collection period. Data on nightly lunar phase was obtained from the Astronomical Applications Department of the United States (http://aa.usno.navy.mil/data).

### Actigraphy

Motionwatch 8 actigraphy watches were worn by each participant during this study. Although polysomnography remains the gold standard for sleep measurement, it is difficult to use in the field primarily because of the invasiveness of the technique—the device is attached to the participant through wired sensors, making it burdensome and difficult to implement in non-laboratory settings^9^. Actigraphy devices are better suited for foraging communities as they are non-invasive and unobtrusive for participants^9^. These devices have been validated against polysomnography for investigating sleep in field environments and allow for larger field sampling, as well^9,51^. All watches were set to record activity data in 1-min epochs, a common duration used to record human actigraphic sleep^9^. These devices have an algorithm that detects and differentiates periods of wakefulness and inactivity. The software then translates these into sleep and wake periods based on a threshold level^9^.

### Statistical analyses

The CamNtech Motionware analysis program was used to score sleep data. The data was then compiled in Microsoft Excel and analyzed in R^52^. Mean averages of BaYaka sleep variables (men and women, both overall and within each location) were compared using Bayes levels of significance (R package *BayesFactor*), controlling for repeated nights of the same subject. Though the current standard of statistical analysis is frequentist testing (which reports rejection of the null using *p* values), a benefit of Bayesian statistical analysis is that Bayes quantifies both the alternative hypothesis (difference between two samples) and the null hypothesis (no difference between two samples)^53^. Bayes testing provides the probability of support for either hypothesis, rather than using a single point estimate to reject the null^53^. A higher, positive Bayes factor provides stronger support for the alternative hypothesis, while a higher negative factor provides stronger support for the null hypothesis. Interpretation of Bayes factor for the alternative hypothesis is adapted from Raftery^54^, using the terms “weak” (Bayes Factor = 1 to 3), “modest” (BF = 3 to 10), “strong” (BF = 10 to 30), and “very strong” (BF > 30)^53^.

Non-Parametric Circadian Rhythm Analysis (NPCRA) was used to assess circadian rhythms between the forest and village, as well as between men and women. In addition, NPCRA analysis calculates the most and least active periods of individuals studied. As seven consecutive nights of sleep data are required for NPCRA analysis, 31 individuals (*n* = 16 village, *n* = 15 forest; n = 16 men, n = 12 women) are represented. Using NPCRA analysis, we measured location and gender differences in interdaily stability, intradaily variability, M10 values, L5 values, and relative amplitude using Bayes factors. Interdaily stability is the similarity of activity patterns from day-to-day, which range from 0 to 1, where 0 indicates no rhythm and 1 is total stability of rhythm. Intradaily variability is the degree of fragmentation in activity-rest periods which ranges from 0 to 2, where higher values indicate higher fragmentation. Most healthy adults will fall < 1. M10 values give the average activity level for the 10 most active hours in a 24-averaged period. M10 indicates how regular and active are the wake periods. L5 values are the least 5 active hours in averaged 24-h periods. L5 indicates how regular the inactive sleep periods are. Relative amplitude is calculated by dividing amplitude by the sum of L5 and M10 and ranges from 0 to 1, with 1 indicating a higher amplitude circadian rhythm. Additionally, the variability of sleep timing (sleep onset/sleep end) was compared between men and women in each location using an F-test of variance.

Four linear mixed effects models were constructed using the *lme4* package in R to test ecological predictors on sleep. Outcome variables for each of these models were (1) total sleep time (TST) defined as the amount of time scored as the main sleep bout in hours, (2) 24-h total sleep time (TTST) defined as the total sleep time across a 24 h period in hours, including sleep and daytime naps, (3) sleep efficiency, defined as the time asleep expressed as a percentage of time in bed, (4) sleep fragmentation, an index of the degree of fragmentation within the sleep period, and measured as the sum of mobile bouts (expressed as a % of inferred sleep time) and immobile bouts $\le$ 1 min (expressed as a % of the total number of immobile bouts). Both sleep efficiency and sleep fragmentation are common quantitative measurements to assess sleep quality. Reference variables in all models were set as *women* for gender and *village* for location. An interaction effect between gender and location was included to test gender-based sleep differences between the village and forest locations. Environmental variables that are known to influence sleep (temperature, rainfall, humidity, and moon phase) were controlled for in the models; these predictor variables were scaled (mean-centered) for comparability with each other. Additionally, we included a random effect for subject to control for repeated night measures. Thus, the following model was used:

$$\begin{aligned} & Sleep \, quota \sim rainfall + temperature + humidity + age \\ & \quad + gender + location + gender*location + (1| {Subject} |) \\ \end{aligned}$$

Using the MultiModel Inference (*MuMIn*) package in R^55^, we averaged linear models with Akaike Information Criterions (AIC) < 10 and reported the importance of each factor within a 95% confidence envelope. To make Bayesian statistical inferences, we then used a shrinkage method to obtain the conditional model averages; shrinkage uses pooled information from more certain estimates of the regression model in order to improve less certain estimates. Pooling, in this instance based off a normal distribution, means that a category provides information that can be used to improve the estimates for all other categories^56^. For our results, we report the standardized coefficients and 95% confidence intervals for each factor.

Lastly, functional linear modelling (FLM) was employed to compare minute-by-minute individual sleep–wake activity patterns across two consecutive days. Two functional linear models were created: one comparing activity patterns in the forest and village locations, and one comparing activity patterns between men and women. FLM is a method developed specifically for actigraphy data that allows time-series data analysis over a 24-h period^57^. FLM uses raw actigraphy counts for each individual to avoid a masking of differences between groups that can sometimes occur with summary statistics^57, 58^. FLM analysis was conducted using a nonparametric permutation test method in R using the package *actigraphy*^59^. A point-wise test (with 500 permutations) was used for the FLM models with a significance level of 0.05.

### Ethical approval

Community consent for this study was obtained at the beginning of the field season. Participants each provided additional informed verbal consent following recruitment into this study. Approval to conduct research in the Republic of Congo was given by The Centre de Recherche et D’Edudes en Sciences Sociales et Humaines. Ethics approval was obtained from Duke University [IRB Protocol # 2017‐0038], the University of Notre Dame for 2017 [IRB Protocol # 17-03-3687], and the University of Cambridge for 2018 [PRE .2018 .023]. The informed consent process and the data collection were conducted based on Duke University, University of Notre Dame, and University of Cambridge ethics guidelines and regulations.

## Data Availability

The datasets generated during the current study are available from the corresponding authors on reasonable request.
